# Efficacy and safety evaluation of first-line systemic treatments for unresectable esophageal squamous cell carcinoma: a network meta-analysis

**DOI:** 10.3389/fonc.2024.1397960

**Published:** 2024-09-09

**Authors:** Huiling Shi, Yong Tan, Chao Ma, Yushan Wei, Fengling Shi, Juan Wang, Caihua Xu, Rongrui Liang

**Affiliations:** ^1^ Department of Oncology, The Fourth Affiliated Hospital of Soochow University, Suzhou, China; ^2^ Division of Clinical Oncology, Medical Center of Soochow University, Suzhou, China; ^3^ School of Physical Education, Soochow University, Suzhou, China; ^4^ Department of Oncology, The First Affiliated Hospital of Soochow University, Suzhou, China

**Keywords:** first-line systemic treatments for esophageal cancer first-line treatment, advanced esophageal squamous carcinoma, network meta-analysis, efficacy, safety

## Abstract

**Objective:**

To evaluate the efficacy and safety of various first-line initial treatment systemic regimens for patients with unresectable esophageal squamous carcinoma(ESCC), utilizing a network meta-analysis approach.

**Methods:**

A comprehensive search for randomized controlled trials focusing on the primary treatment of esophageal cancer ESCC was conducted across multiple databases including PubMed, Embase, Cochrane Library, and Web of Science, up until November 17, 2023. The quality of the included studies was rigorously assessed using Review Manager software. Subsequently, data analysis was meticulously carried out employing R software. The first-line treatment regimens examined were: CD (Cisplatin + Docetaxel), CET-CF (Cetuximab + Cisplatin + Fluorouracil), CF (Cisplatin + Fluorouracil), N-CF (Nivolumab + Cisplatin + Fluorouracil), NI (Nivolumab + Ipilimumab), Nim-CF (Nimotuzumab + Cisplatin + Fluorouracil), P-CF (Pembrolizumab + Cisplatin + Fluorouracil), and Ser-CF (Serplulimab + Cisplatin + Fluorouracil). The Primary endpoints included the overall survival(OS),progression-free survival (PFS),objective response rate (ORR) and disease control rate (DCR).The secondary endpoint was adverse effects(AEs).

**Results:**

The analysis encompassed eight studies, incorporating a total of 3,051 patients with untreated esophageal cancer. There are 45 people in the CD regimen,32 in the CET-CF regimen,1,212 in the CF regimen,447 in the N-CF regimen,456 in the NI regimen,53 in the Nim-CF regimen,447 in the P-CF regimen and 368 in the Ser-CF regimen. The network meta-analysis revealed that, in comparison to the CF regimen, the other regimens (CD, CET-CF, N-CF, NI, Nim-CF, P-CF, and Ser-CF) did not demonstrate a statistically significant impact on overall survival (OS) or progression-free survival (PFS). However, Ser-CF potentially offers superior outcomes in terms of OS and PFS when juxtaposed with other regimens. Notably, N-CF was associated with a substantial increase in the objective response rate (ORR), and CET-CF markedly improved the disease control rate (DCR). In terms of adverse effects, N-CF was more likely to cause anorexia, whereas CET-CF was significantly associated with nausea, vomiting, neutropenia, and skin disorders.

**Conclusion:**

The current evidence suggests that N-CF may provide the most favorable outcomes in terms of ORR, while CET-CF could be the optimal choice for enhancing DCR in patients with untreated esophageal cancer.

## 1 Introduction

Esophageal cancer is recognized as a highly lethal malignancy with a rapidly rising global incidence. Notably, it stands as the fourth most common cause of cancer-related mortality worldwide, showcasing a 5-year survival rate between 15% and 25%. The disease poses a significant challenge to public health. Predominantly, esophageal cancer manifests in two primary histological forms: esophageal adenocarcinoma (EADC) and esophageal squamous cell carcinoma (ESCC), with the latter being more common (1). Particularly in developed nations, EADC tends to be the prevalent diagnosis among esophageal cancer patients. Contrastingly, in China, ESCC accounts for over 90% of cases, especially prevalent in the northern Taihang Mountains region, where it stands as the leading cause of death (2).

The development of ESCC is closely linked with factors such as low socioeconomic status, consumption of tobacco and alcohol, ingestion of hot beverages, and exposure to nitrosamines. Additionally, insufficient levels of micronutrients like vitamin C, vitamin E, and folate are also implicated in ESCC risk. Conversely, risk factors for EADC include conditions like Barrett’s esophagus, gastroesophageal reflux disease (GERD), obesity, and tobacco use (3). Esophageal cancer ESCC is known for its aggressive nature and tendency to be diagnosed at advanced stages due to the typically late emergence of symptoms. For years, the CF regimen (Cisplatin + fluorouracil) was the standard treatment, yet its efficacy in tumor suppression is suboptimal (4), the prevalence of esophageal cancer remains high (5). Recent years have witnessed significant strides in cancer treatment, particularly with the advent of targeted therapies and immunotherapies. Notably, the effectiveness of pembrolizumab in combination with chemotherapy over chemotherapy alone has been documented (6). Moreover, positive outcomes from the Checkmate 649 study have positioned Nivolumab combined with chemotherapy as a frontline therapy for advanced EADC (7). Additionally, the combination of Nivolumab and Ipilimumab (NI) has received approval for treating advanced ESCC patients (8).

Presently, a variety of first-line treatment options are available, including CF, CD, CET-CF, N-CF, NI, Nim-CF, P-CF, and Ser-CF. However, the absence of direct comparisons among these treatments and the urgent need to identify the most effective approach have led us to pursue a meta-analysis. Our endeavor aims to clarify the debate over the optimal treatment regimen, potentially offering novel references for future clinical applications.

## 2 Methods and materials

### 2.1 Literature search

A systematic review was performed to assess the effectiveness and safety of first-line treatments for esophageal cancer. This review entailed a computer-based search of randomized controlled trials (RCTs) within the Cochrane, PubMed, Embase, and Web of Science databases, culminating on November 17, 2023. The search methodology incorporated a blend of controlled vocabulary terms and free-text keywords including “esophageal cancer,” “newly diagnosed,” “untreated,” “first-line,” “front-line,” and “initial.” The specifics of the search strategies are delineated in **Suplementary Table S1**.

### 2.2 Inclusion and exclusion criteria

Inclusion Criteria: This review rigorously selected studies involving adult patients who have been diagnosed with esophageal cancer. The interventions scrutinized included Cisplatin and Docetaxel (CD), Cisplatin, Epirubicin, and Capecitabine (CET-CF), Nedaplatin and Capecitabine (N-CF), Nedaplatin and Irinotecan (NI), Nimotuzumab and Capecitabine (Nim-CF), Paclitaxel and Capecitabine (P-CF), and S-1 and Capecitabine (Ser-CF), compared against a control group receiving Capecitabine (CF) alone. The primary objectives of this review were to assess overall survival (OS), progression-free survival (PFS), the objective response rate (ORR), and the disease control rate (DCR). Secondary outcomes were focused on the evaluation of adverse events. Only studies designed as randomized controlled trials (RCTs) met the criteria for inclusion.

Exclusion Criteria: The review excluded studies if they were redundant publications, animal research, case reports, conference abstracts, or reviews. Furthermore, studies were excluded if they included patients with concurrent organic diseases.

### 2.3 Data extraction

The selection of studies was meticulously undertaken by two authors, who independently applied the pre-established inclusion and exclusion criteria. Any discrepancies that arose during this process were amicably resolved through mutual discussion between the authors. If a consensus could not be reached, the matter was escalated to a third, neutral party for arbitration. The data harvested from the studies that met these criteria encompassed critical variables, including but not limited to the primary author’s name, the year of publication, the country of origin, the sample size, the distribution of participants by gender, their ages, the ECOG (Eastern Cooperative Oncology Group) performance status, the nature of the interventions administered, and the metrics used to gauge the outcomes.

### 2.4 Quality assessment

The evaluation of bias risk was conducted in accordance with the most current guidelines stipulated by the Cochrane Risk of Bias Assessment Tool 2.0 (ROB 2.0) (9). This comprehensive tool outlines five critical domains to scrutinize: bias emerging from the randomization process, bias resulting from deviations from the intended interventions, bias associated with missing outcome data, bias in the measurement of outcomes, and bias in the selection of reported results. The assessment of quality categorized the studies into three distinct levels: “low risk of bias,” “some concerns,” or “high risk of bias.” To ensure reliability, the findings were independently verified by two reviewers. Any inconsistencies identified during this process were meticulously addressed, either by reaching a consensus through discussion or, if necessary, by seeking the judgment of a third party.

### 2.5 Data analysis

A Bayesian network meta-analysis was performed utilizing R software version 4.2.3 (R Foundation for Statistical Computing) (10, 11), adopting *a priori* vague random-effects models. The analysis employed Markov chain Monte Carlo techniques, as described by (12), to derive optimal combined estimates and associated probabilities for each treatment option. For representing survival data, hazard ratios (HRs) and their 95% credible intervals (CIs) were used, while binary outcomes were depicted through odds ratios (ORs) and their 95% CIs.

The Surface Under the Cumulative Ranking curve (SUCRA) was calculated to gauge the likelihood of each intervention being the most effective. Network and funnel plots were generated using STATA version 15.0, utilizing a direct macro command for their creation. In the network plot, drugs are represented by circles, with the size of each circle being proportional to the number of patients included in the respective trials. The links between circles (edges) denote the direct comparisons available between drugs. Furthermore, cumulative probability distributions were visualized using the ggplot2 package in R.

## 3 Results

### 3.1 Data screening process and results

**Figure 1:**
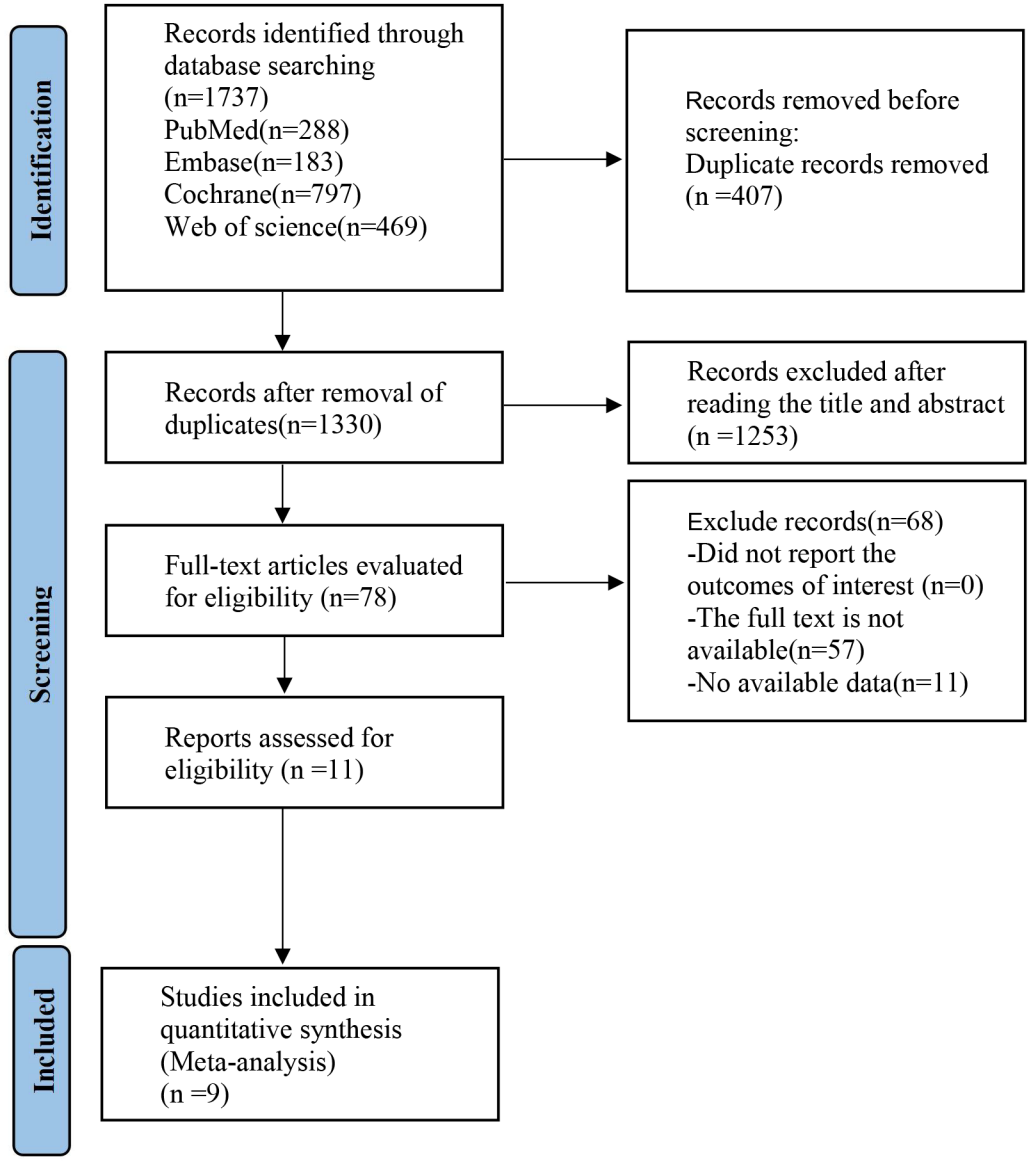
Literature search flowchart.

An initial search of the databases retrieved a total of 1,737 articles. After the elimination of duplicates, this number was reduced to 407. A detailed screening of titles and abstracts led to the exclusion of 1,263 articles. Further scrutiny through full-text review resulted in the removal of 69 more articles. Ultimately, eight articles (13–20) were selected for in-depth analysis, as depicted in **Figure 1**.

### 3.2 Basic characteristics of included studies and risk of bias assessment

**Figure 2:**
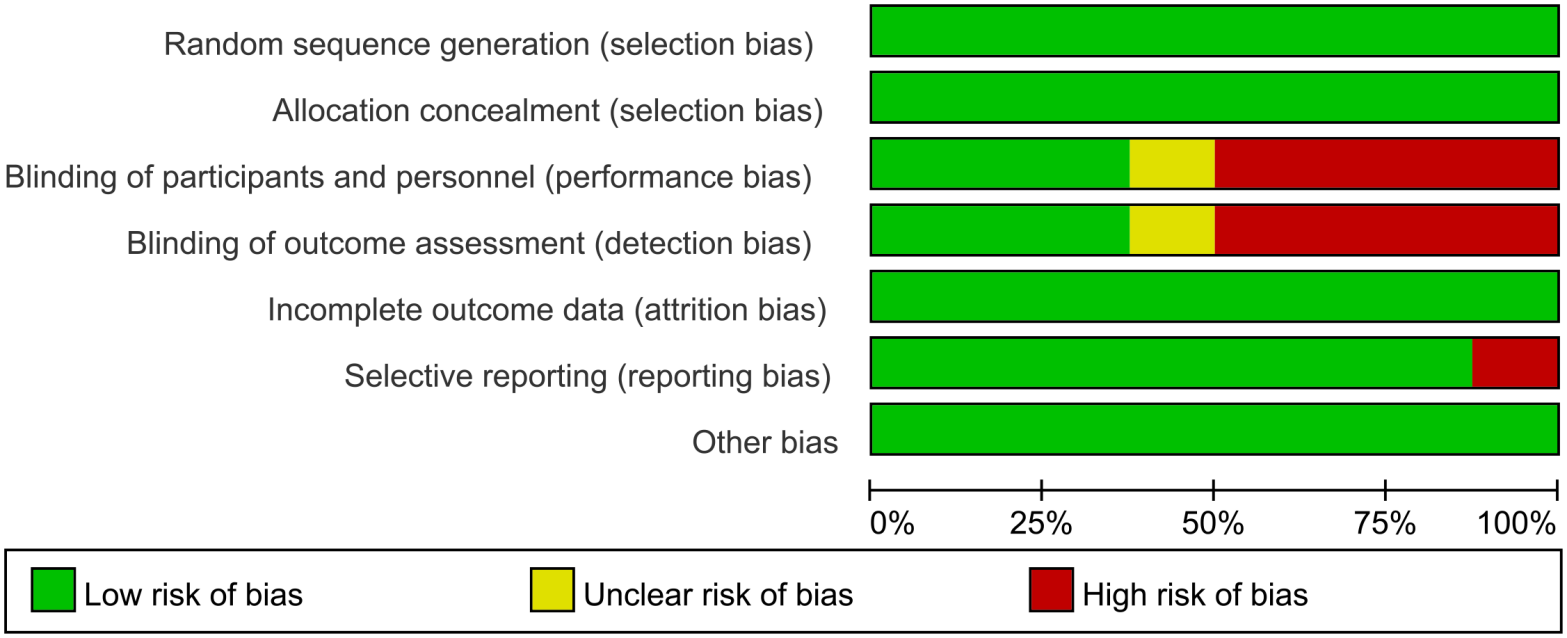
Risk of bias graph.

**Figure 3:**
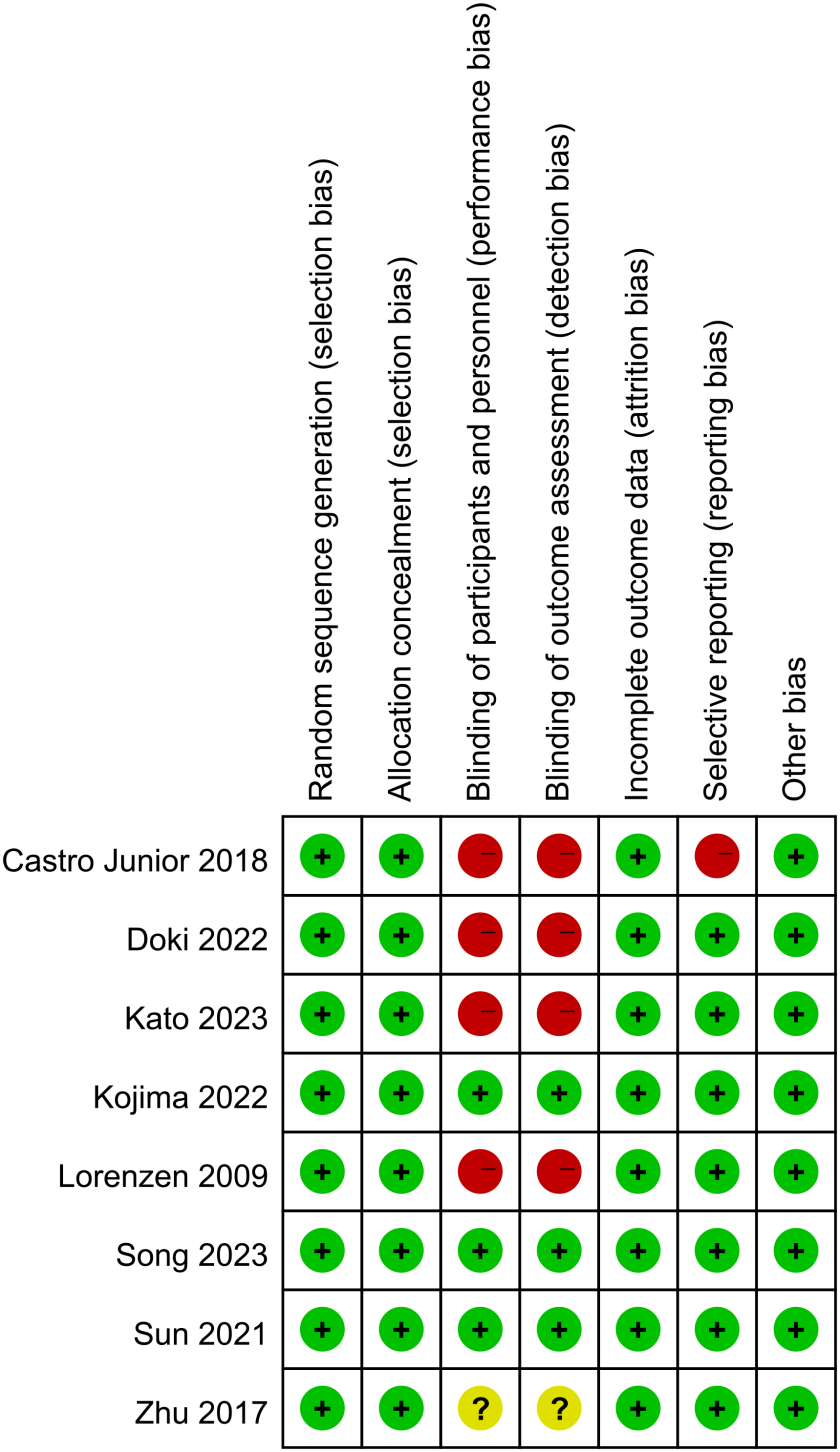
Risk of bias summary.

The analysis encompassed eight articles, comprising a total of 3051 untreated esophageal cancer patients. Specific characteristics of the included studies can be found in **Table 1**. All studies included in this analysis clearly described their blinding methods. High risk primarily stemmed from deviations in the expected intervention measures, and the risk of bias assessment for the included studies can be found in **Figures 2** and **3**.

**Table 1:** Literature characteristics table.

Study	Year	Country	Sample size	Gender(M/F)	Mean age(years)	ECOG	intervention	Follow-up	outcome	Median os(Mo)	Median Pfs(Mo)
Lorenzen	2009	Germany	CF:30 CET-CF:32	29/1 23/9	CF:62 CET-CF:61	(0/1):15/15 (0/1):17/15	CF:iv;cisplatin(100mg/m^2)^+5-fluorouracil(1000mg/m^2^); cycle/29days CET-CF:iv;cisplatin(100mg/m^2^)+5-fluorouracil(1000mg/m^2^)+cetuximab(initial400mg/m^2^,followed250mg/m^2^);cycle/29days	24month	OS; PFS; ORR; DCR; AEs	CF:5.5 CET-CF:9.5	CF:3.6 CET-CF:5.9
Zhu	2017	China	CF:41 CD:45	29/12 31/14	CF:59 CD:58		CF:iv;Cisplatin(80mg/m^2^)+5-fluorouracil(1000mg/m^2^);cycle/3 week CD:iv;Cisplatin(80mg/m^2^)+docetaxel(60mg/m^2^);cycle/3 week	60month	ORR; DCR; OS; PFS; AEs	NR	NR
Castro Junior	2018	Brazil	Nim-CF:53 CF:54	44/9 44/10	Nim-CF:60 CF:58.5	(0/1/2):21/31/1 (0/1/2):15/33/6	Nim-CF:iv;Cisplatin(75mg/m^2^)+fluorouracil(1000mg/m^2^,d1-d4)+nimotuzumab(200mg);cycle/4 week CF:iv;Cisplatin(75mg/m^2^)+5-fluorouracil(1000mg/m^2^,d1-d4);cycle/4 week	50month	OS; AEs	Nim-CF:15.9 CF:11.5	NR
Sun	2021	South Korea	P-CF:373 CF:376	306/67 319/57	P-CF:64 CF:62	(0/1/2):149/223/1 (0/1/2):150/225/1	P-CF:iv;Pembrolizumab(200mg)+Cisplatin(80mg/m^2^)+5-fluorouracil(800mg/m^2^,d1-5);cycle/3 week CF:placebo+Cisplatin(80mg/m^2^)+5-fluorouracil(800mg/m^2^,d1-5);cycle/3 week	36month	OS; PFS; ORR; AEs	P-CF:12.4 CF:9.8	P-CF:6.3 CF:5.8
Doki	2022	Japan	N-CF:321 NI:325 CF:324	253/68 269/56 275/49	(0/1):150/171 (0/1):151/174 (0/1):154/179	N-CF:64 NI:63 CF:64	N-CF:iv;Nivolumab(240mg,cycle/2week)+Cisplatin(80mg/m^2^)+fluorouracil(800mg/m^2^,d1-5);cycle/4 week NI:iv;Nivolumab(3mg/kg,cycle/2week)+Ipilimumab(1mg/kg,cycle/6week) CF: Cisplatin(80mg/m^2^)+fluorouracil(800mg/m^2^,d1-5);cycle/4 week	42month	OS; PFS; ORR; DCR; AEs	N-CF:13.2 NI:12.7 CF:10.7	N-CF:5.8 NI:2.9 CF:5.6
Kojima	2022	Japan	P-CF:74 CF:67	63/11 61/3	(0/1):48/26 (0/1):53/14	P-CF:68 CF:68	P-CF:iv;Pembrolizumab(200mg)+Cisplatin(80mg/m^2^)+5-fluorouracil(800mg/m^2^);cycle/3 week CF:placebo+Cisplatin(80mg/m^2^)+5-fluorouracil(800mg/m^2^);cycle/3 week	36month	OS; PFS; ORR; AEs	P-CF:17.6 CF:11.7	P-CF:6.3 CF:6.0
Kato	2023	Japan	NI:131 N-CF:126 CF:137	111/20 99/27 121/16	(0/1):93/38 (0/1):89/37 (0/1):95/42	NI:66 N-CF:68 CF:67	NI:iv;Nivolumab(3mg/kg,cycle/2week)+Ipilimumab(1mg/kg,cycle/6week) N-CF:iv;Nivolumab(240mg,cycle/2week)+Cisplatin(80mg/m^2^)+fluorouracil(800mg/m^2^,d1-5);cycle/4 week CF: Cisplatin(80mg/m^2^)+fluorouracil(800mg/m^2^,d1-5);cycle/4 week	42month	OS; PFS; ORR; AEs	NI:17.6 N-CF:15.5 CF:11.0	NI:4.2 N-CF:6.8 CF:4.3
Song	2023	China	Ser-CF:368 CF:183	317/51 153/30	(0/1):93/275 (0/1):53/130	Ser-CF:64 CF:64	Ser-CF:iv;Serplulimab(3mg/kg)+Cisplatin(50mg/m^2^)+5-fluorouracil(1200mg/m^2^,d1,d2);cycle/2week CF:placebo+Cisplatin(50mg/m^2^)+5-fluorouracil(1200mg/m^2^,d1,d2);cycle/2week	34month	OS; PFS; ORR; DCR; AEs	Ser-CF:15.3 CF:11.8	Ser-CF:5.8 CF:5.3

M/F, Male/Female; CF, Cisplatin + 5-fluorouracil; P-CF, Pembrolizumab+Cisplatin + 5-fluorouracil; N-CF, Nivolumab+Cisplatin+fluorouracil; Nim-CF, Cisplatin+fluorouracil+nimotuzumab; Ser-CF, Serplulimab+Cisplatin + 5-fluorouracil; CET-CF, Cetuximab+cisplatin+ 5-fluorouracil; NI, Nivolumab+Ipilimumab; CD, Cisplatin+docetaxel; ORR, Objective response rate; DCR, Disease control rate; OS, Overall survival; PFS, Progression-free survival; AEs, Adverse events.

### 3.3 Network meta-analysis results

#### 3.3.1 Overall survival

**Figure 4:**
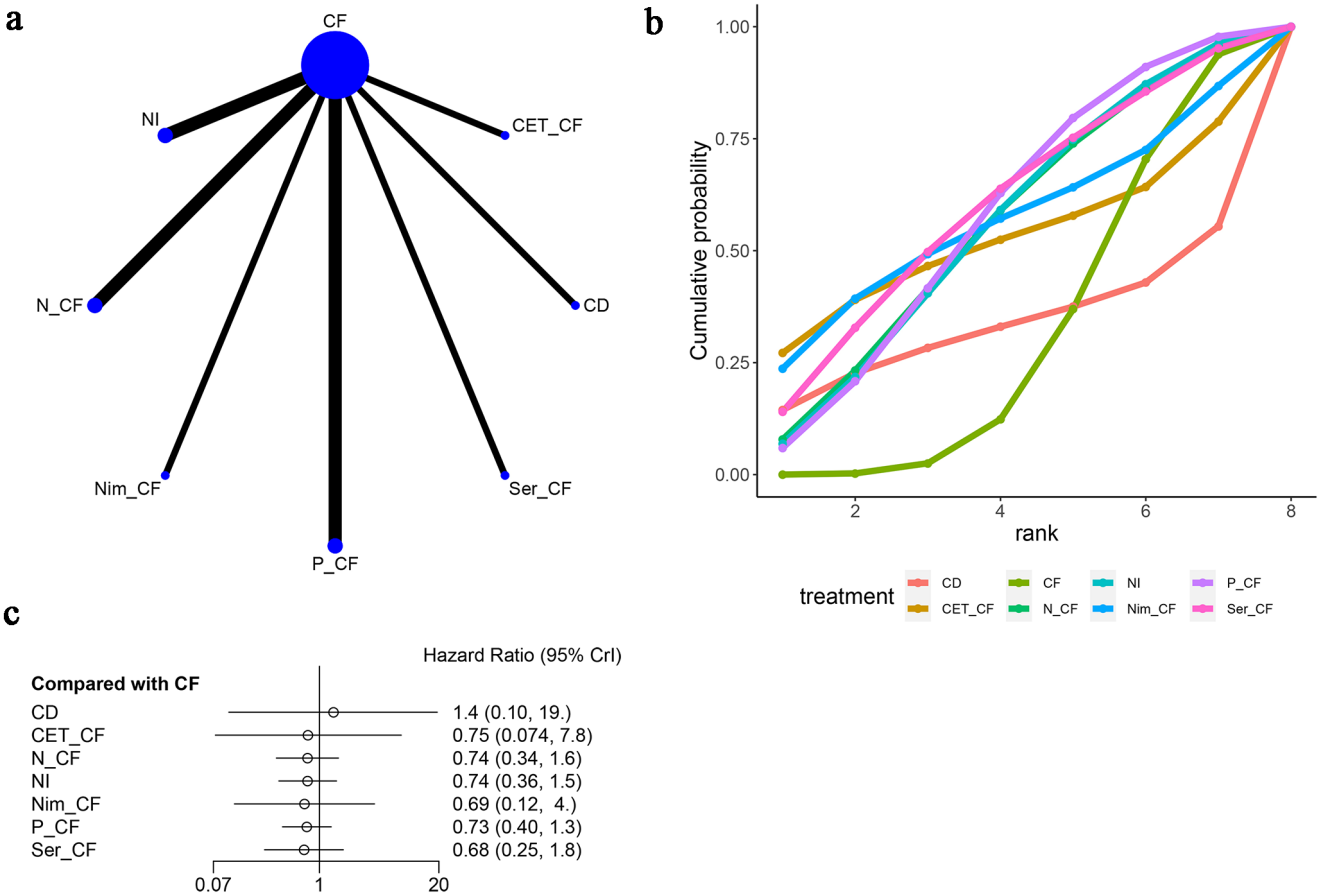
Meta-Analysis of Overall Survival (OS). **(A)** Network Plot(Each circle represents a different intervention, with the size of the circle proportional to the number of people in that intervention, and the line between the circles represents the existence of a direct comparison between the two interventions, with the thickness of the line representing the proportional number of studies), **(B)** Area under the Cumulative Probability Curve(Surface Under the Cumulative Ranking Curve of different intervention, CD, Cisplatin + Docetaxel; CF, Cisplatin + Fluorouracil; NI, Nivolumab + Ipilimumab,P-CF, Pembrolizumab + Cisplatin + Fluorouracil,CET-CF, Cetuximab + Cisplatin + Fluorouracil,N-CF, Nivolumab + Cisplatin + Fluorouracil,Nim-CF, Nimotuzumab + Cisplatin + Fluorouracil,Ser-CF, Serplulimab + Cisplatin + Fluorouracil); **(C)** Forest Plot.

In a network meta-analysis comprising eight studies (13–20), overall survival (OS) outcomes were evaluated (refer to **Figure 4**). The network graph depicted in **Figure 4A** indicated the absence of closed loops, allowing for direct comparisons among various treatment regimens: CF with NI, N-CF, Nim-CF, P-CF, Ser-CF, CD, and CET-CF.

When juxtaposed with CF, the hazard ratios (HRs) for each treatment were as follows: for CD, HR=1.42 with a 95% confidence interval (CI) of (0.1, 19.19); for CET-CF, HR=0.75, CI (0.074, 7.8); for N-CF, HR=0.74, CI (0.34, 1.6); for NI, HR=0.74, CI (0.36, 1.5); for Nim-CF, HR=0.69, CI (0.12, 4.0); for P-CF, HR=0.73, CI (0.40, 1.3); and for Ser-CF, HR=0.68, CI (0.25, 1.8). Despite these findings, none of the interventions demonstrated a significant impact on OS, as illustrated in **Figure 4C**, and no notable differences were observed among the various first-line treatment regimens (**Table 2A**).

Table 2League Table.

**Table:** 

a. OS League Table							
HR (95%Crl)							
CD							
1.89 (0.06, 62.6)	CET_CF						
1.42 (0.1, 19.19)	0.75 (0.07, 7.78)	CF					
1.92 (0.13, 29.35)	1.02 (0.09, 12.01)	1.35 (0.62, 2.94)	N_CF				
1.91 (0.12, 28.6)	1.01 (0.09, 11.65)	1.34 (0.65, 2.76)	0.99 (0.34, 2.87)	NI			
2.06 (0.09, 47.39)	1.09 (0.06, 20.44)	1.45 (0.25, 8.41)	1.07 (0.16, 7.31)	1.08 (0.16, 7.16)	Nim_CF		
1.94 (0.13, 28.32)	1.03 (0.09, 11.47)	1.37 (0.75, 2.52)	1.01 (0.38, 2.72)	1.02 (0.4, 2.63)	0.95 (0.15, 6.06)	P_CF	
2.08 (0.13, 33.94)	1.1 (0.09, 13.97)	1.47 (0.55, 3.95)	1.09 (0.31, 3.83)	1.09 (0.32, 3.73)	1.02 (0.14, 7.59)	1.07 (0.34, 3.43)	Ser_CF

**Table:** 

b. PFS League Table						
HR(95%Crl)						
CD						
1.24 (0.05, 30.55)	CET_CF					
1.35 (0.12, 15.18)	1.08 (0.13, 8.75)	CF				
1.7 (0.13, 21.59)	1.37 (0.15, 12.68)	1.26 (0.59, 2.69)	N_CF			
1.09 (0.09, 13.42)	0.88 (0.1, 7.92)	0.81 (0.43, 1.55)	0.65 (0.24, 1.75)	NI		
2.12 (0.18, 25.67)	1.71 (0.19, 15)	1.57 (0.87, 2.85)	1.25 (0.47, 3.28)	1.93 (0.8, 4.66)	P_CF	
2.24 (0.17, 29.64)	1.81 (0.19, 17.52)	1.67 (0.68, 4.06)	1.32 (0.41, 4.28)	2.05 (0.68, 6.16)	1.06 (0.36, 3.09)	Ser_CF

**Table:** 

c. ORR League Table						
OR(95%Crl)						
CD						
0.59 (0.11, 3.17)	CET_CF					
0.74 (0.2, 2.59)	1.23 (0.42, 3.7)	CF				
0.26 (0.07, 0.93)	0.43 (0.14, 1.34)	0.35 (0.27, 0.46)	N_CF			
0.6 (0.16, 2.18)	1.01 (0.33, 3.15)	0.82 (0.61, 1.09)	2.33 (1.56, 3.48)	NI		
0.37 (0.1, 1.33)	0.62 (0.2, 1.92)	0.5 (0.38, 0.66)	1.43 (0.96, 2.11)	0.61 (0.41, 0.91)	P_CF	
0.39 (0.1, 1.43)	0.65 (0.21, 2.06)	0.53 (0.37, 0.75)	1.5 (0.95, 2.36)	0.64 (0.41, 1.02)	1.05 (0.67, 1.65)	Ser_CF

**Table:** 

d. DCR League Table					
OR(95%Crl)					
CD					
0.19 (0.01, 2.99)	CET_CF				
0.45 (0.01, 5.7)	2.35 (0.8, 7.26)	CF			
0.3 (0.01, 3.91)	1.58 (0.52, 5.11)	0.68 (0.49, 0.92)	N_CF		
0.81 (0.03, 10.57)	4.3 (1.43, 13.77)	1.83 (1.39, 2.43)	2.71 (1.79, 4.15)	NI	
0.36 (0.01, 4.76)	1.89 (0.59, 6.32)	0.8 (0.52, 1.25)	1.19 (0.7, 2.04)	0.44 (0.26, 0.74)	Ser_CF

Grey zone: League tables for each major outcomes. Blue zone: Type of intervention. Green zone: Comparison between two interventions.

Notably, Ser-CF emerged as a potentially superior option, achieving the highest score on the area under the cumulative ranking curve (59.5%), followed by P-CF (57.1%), Nim-CF (56.1%), and CF (31.0%) as depicted in **Figure 4B** and **Table 3**.

**Table 3:** SUCRA comprehensive ranking.

	OS (%)	PFS (%)	ORR (%)	DCR (%)	NEU (%)	NAU (%)	SKIN (%)	ANO (%)
CD	33.5	37.0	84.0	75.8	/	/	/	/
CET_CF	52.3	43.0	60.2	10.7	0.90	5.0	0.00	/
CF	31.0	38.9	81.3	60.8	73.6	59.6	98.7	57.6
N_CF	55.4	57.4	2.83	24.7	43.9	25.1	45.5	33.3
NI	55.2	27.0	62.9	88.6	100	100	18.9	54.4
Nim_CF	56.1	/	/	/	34.6	76.3	48.6	62.1
P_CF	57.1	73.3	27.5	/	36.9	31.7	73.2	40.4
Ser_CF	59.5	73.5	31.4	39.4	60.2	52.2	65.0	52.3

CF, Cisplatin + 5-fluorouracil; P-CF, Pembrolizumab+ Cisplatin + 5-fluorouracil; N-CF, Nivolumab+ Cisplatin+ fluorouracil; Nim-CF, Cisplatin+ fluorouracil+ nimotuzumab; Ser-CF, Serplulimab+ Cisplatin + 5-fluorouracil; CET-CF, Cetuximab+ cisplatin+ 5-fluorouracil; NI, Nivolumab+ Ipilimumab; CD, Cisplatin+ docetaxel; ORR, Objective response rate; DCR, Disease control rate; OS, Overall survival; PFS, Progression-free survival; NEU, Neutropenia; NAU, Nausea; SKIN, Skin disorders; ANO, Anorexic.

### 3.4 Progression-free survival

**Figure 5:**
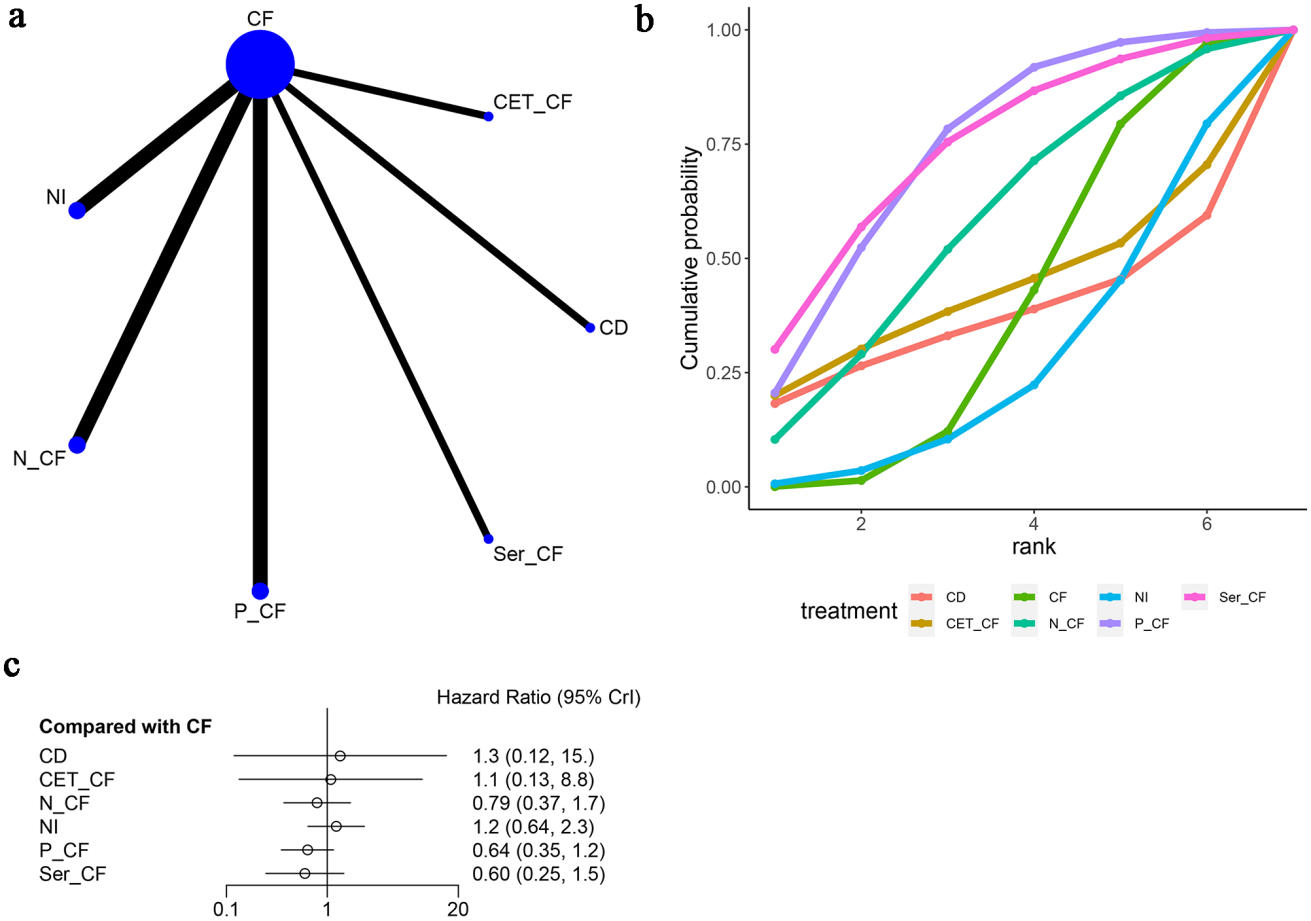
Meta-Analysis of Progression-Free Survival (PFS). **(A)** Network Plot(Each circle represents a different intervention, with the size of the circle proportional to the number of people in that intervention, and the line between the circles represents the existence of a direct comparison between the two interventions, with the thickness of the line representing the proportional number of studies), **(B)** Area under the Cumulative Probability Curve (Surface Under the Cumulative Ranking Curve of different intervention, CD, Cisplatin + Docetaxel; CF, Cisplatin + Fluorouracil; NI, Nivolumab + Ipilimumab,P-CF, Pembrolizumab + Cisplatin + Fluorouracil,CET-CF, Cetuximab + Cisplatin + Fluorouracil,N-CF, Nivolumab + Cisplatin + Fluorouracil,Ser-CF, Serplulimab + Cisplatin + Fluorouracil), **(C)** Forest Plot.

Seven studies (13, 14, 16–20) evaluated progression-free survival (PFS), as depicted in **Figure 5**. The network diagram (**Figure 5A**) illustrates direct comparisons among various treatment combinations: CF with NI, N-CF, P-CF, Ser-CF, CD, and CET-CF. When compared to CF, the hazard ratios (HRs) were as follows: for CD, HR = 1.35 with a 95% confidence interval (CI) of (0.12, 15.18); for CET-CF, HR = 1.08, 95% CI (0.13, 8.8); for N-CF, HR = 0.79, 95% CI (0.37, 1.7); for NI, HR = 1.2, 95% CI (0.64, 2.3); for P-CF, HR = 0.64, 95% CI (0.35, 1.2); and for Ser-CF, HR = 0.60, 95% CI (0.25, 1.5). These treatments showed marginal differences in enhancing PFS (**Table 2B**). Notably, Ser-CF achieved the highest area under the cumulative ranking curve at 73.5%, followed closely by P-CF at 73.3%, N-CF at 57.4%, and NI at 27.0% (**Figure 5B**; **Table 3**).

### 3.5 Objective response rate

**Figure 6:**
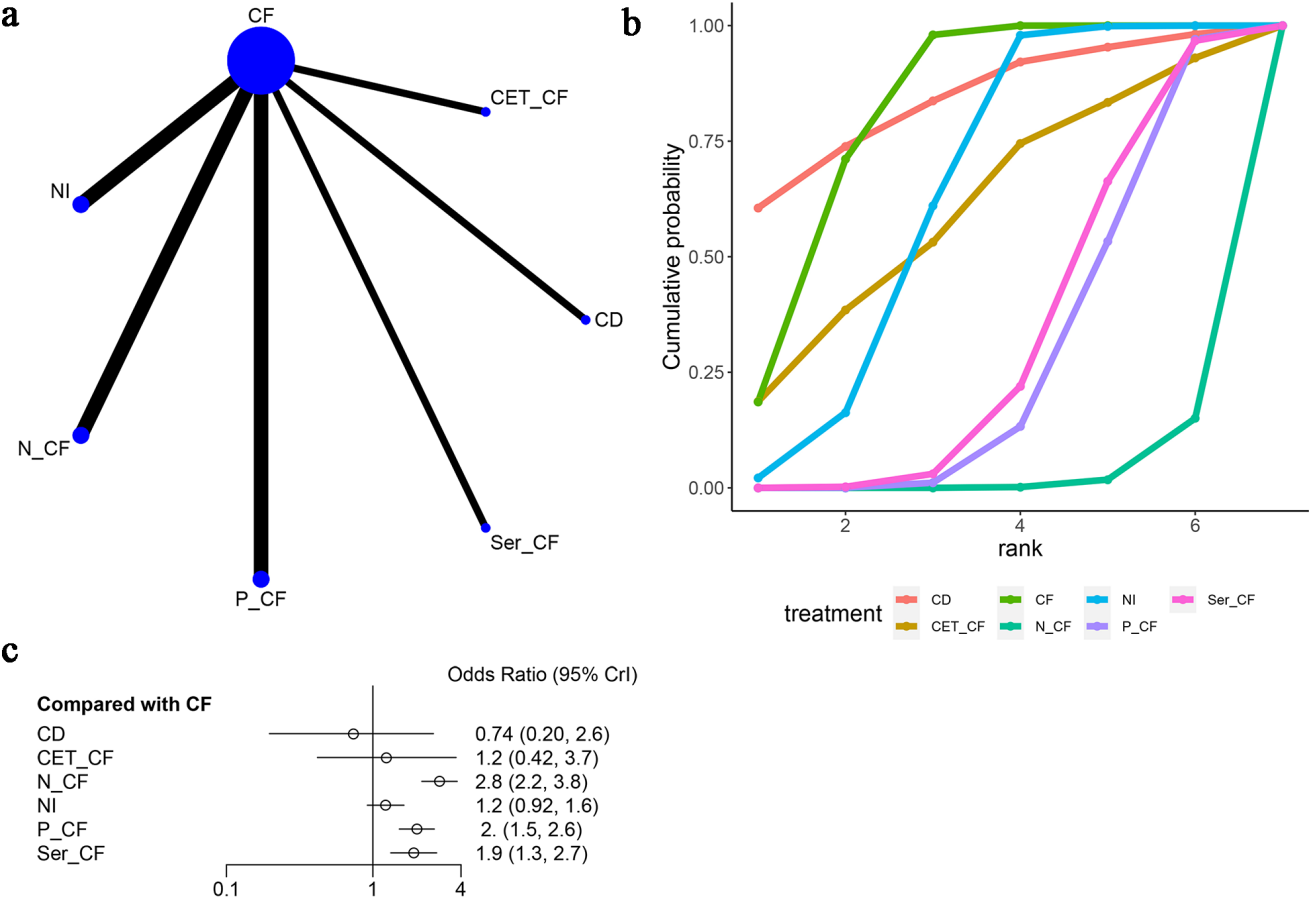
Meta-Analysis of Objective Response Rate (ORR). **(A)** Network Plot(Each circle represents a different intervention, with the size of the circle proportional to the number of people in that intervention, and the line between the circles represents the existence of a direct comparison between the two interventions, with the thickness of the line representing the proportional number of studies), **(B)** Area under the Cumulative Probability Curve (Surface Under the Cumulative Ranking Curve of different intervention, CD, Cisplatin + Docetaxel; CF, Cisplatin + Fluorouracil; NI, Nivolumab + Ipilimumab,P-CF, Pembrolizumab + Cisplatin + Fluorouracil,CET-CF, Cetuximab + Cisplatin + Fluorouracil,N-CF, Nivolumab + Cisplatin + Fluorouracil,Ser-CF, Serplulimab + Cisplatin + Fluorouracil), **(C)** Forest Plot.

In this analysis, seven studies (13, 14, 16–20) were examined to assess the objective response rate (ORR), as depicted in **Figure 6**. The network diagram (**Figure 6A**) delineated direct comparisons among various treatments: Cetuximab and Fluoropyrimidine (CF) versus Nitroglycerin Patch (NI), Non-Cetuximab and Fluoropyrimidine (N-CF), Preoperative Cetuximab and Fluoropyrimidine (P-CF), Serotonin and Cetuximab and Fluoropyrimidine (Ser-CF), Cetuximab and Docetaxel (CD), and Cetuximab monotherapy (CET-CF).

The analysis of odds ratios (ORs) revealed varying effectiveness of treatments compared to CF. Specifically, CD exhibited an OR of 0.74 [95% CI (0.20, 2.6)], indicating less effectiveness, while CET-CF showed an OR of 1.2 [95% CI (0.42, 3.7)], suggesting a marginal improvement. Notably, N-CF [OR=2.8, 95% CI (2.2, 3.8)], NI [OR=1.2, 95% CI (0.92, 1.6)], P-CF [OR=2.0, 95% CI (1.5, 2.6)], and Ser-CF [OR=1.9, 95% CI (1.3, 2.7)] demonstrated more promising results, with N-CF, P-CF, and Ser-CF notably enhancing ORR, as highlighted in **Figure 6C**.

However, treatments such as CET-CF, NI, and CD did not significantly improve ORR, as detailed in **Table 2C**. Among the analyzed treatments, N-CF emerged as the most favorable in enhancing ORR. In terms of the cumulative ranking curve, CD achieved the highest area under the curve (AUC) of 84%, followed by CF (81.3%), NI (62.9%), and N-CF (2.83%), as shown in **Figure 6B** and **Table 3**. This comprehensive analysis underscores the varied efficacy of the treatments, with N-CF standing out for its potential in improving ORR.

#### 3.5.1 DCR

**Figure 7:**
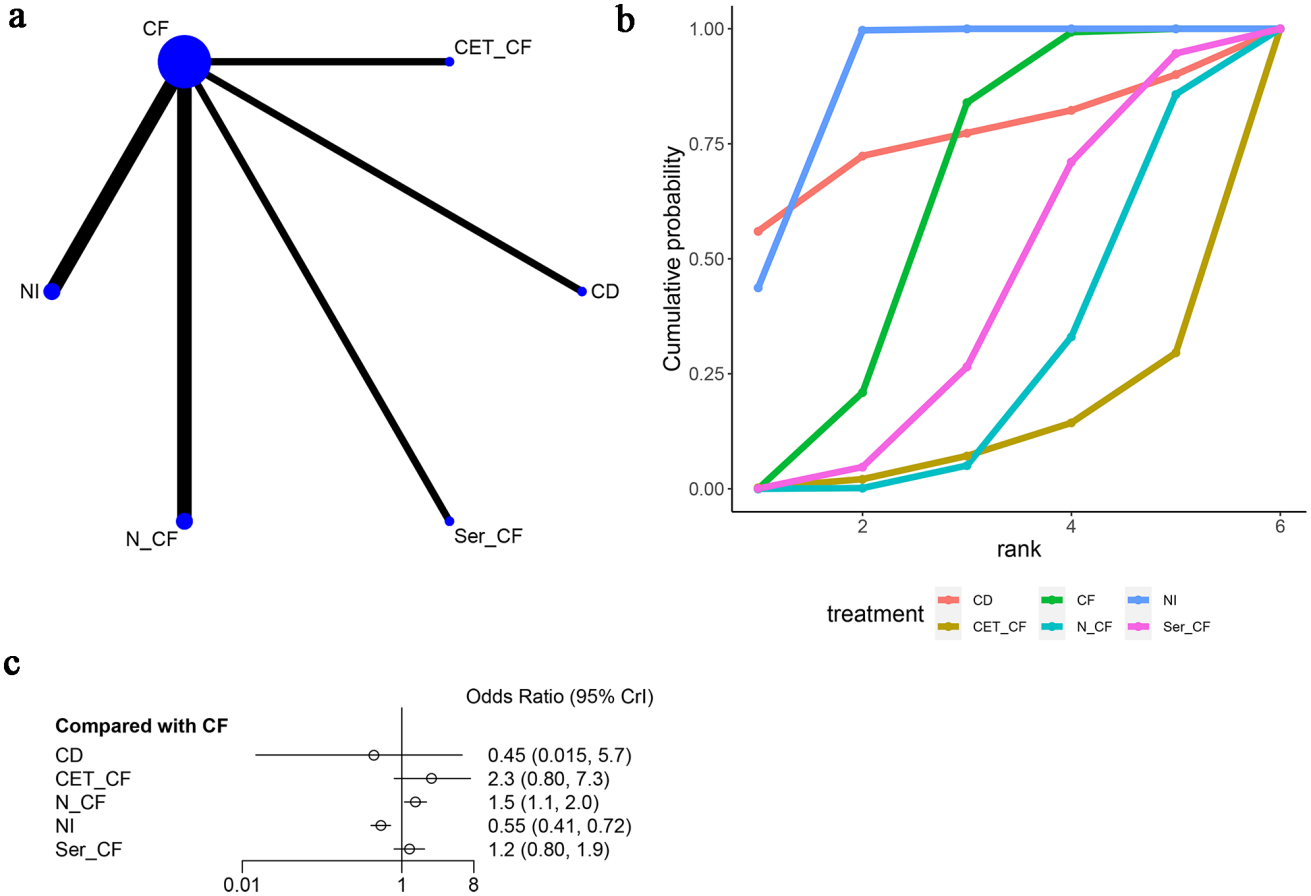
Meta-Analysis of Disease Control Rate (DCR). **(A)** Network Plot(Each circle represents a different intervention, with the size of the circle proportional to the number of people in that intervention, and the line between the circles represents the existence of a direct comparison between the two interventions, with the thickness of the line representing the proportional number of studies), **(B)** Area under the Cumulative Probability Curve(Surface Under the Cumulative Ranking Curve of different intervention, CD, Cisplatin + Docetaxel; CF, Cisplatin + Fluorouracil; NI, Nivolumab + Ipilimumab; CET-CF, Cetuximab + Cisplatin + Fluorouracil,N-CF, Nivolumab + Cisplatin + Fluorouracil,Ser-CF, Serplulimab + Cisplatin + Fluorouracil), **(C)** Forest Plot.

In five articles (13, 14, 17, 19, 20), DCR was mentioned as depicted in **Figure 7**. The network diagram (**Figure 7A**) reveals direct comparisons between CF and NI, CF and N-CF, CF and Ser-CF, CF and CD, as well as CF and CET-CF. Compared to CF, CD [OR=0.45, 95% CI (0.01, 5.7)], CET-CF [OR=2.35, 95% CI (0.8, 7.3)], N-CF [OR=1.5, 95% CI (1.1, 2.0)], NI [OR=0.55, 95% CI (0.41, 0.72)], and Ser-CF [OR=1.2, 95% CI (0.80, 1.9)] were evaluated. In comparison to CF, it was found that CET-CF and N-CF could improve DCR, whereas Ser-CF, CD, and NI did not exhibit a significant improvement in DCR (**Figure 7C**) (**Table 2D**). The area under the cumulative ranking curve indicated that NI had the highest impact (88.6%), followed by CD (75.8%), CF (60.8%), and CET-CF (10.7%) (**Figure 7B**; **Table 3**).

### 3.6 Adverse events

**Figure 8:**
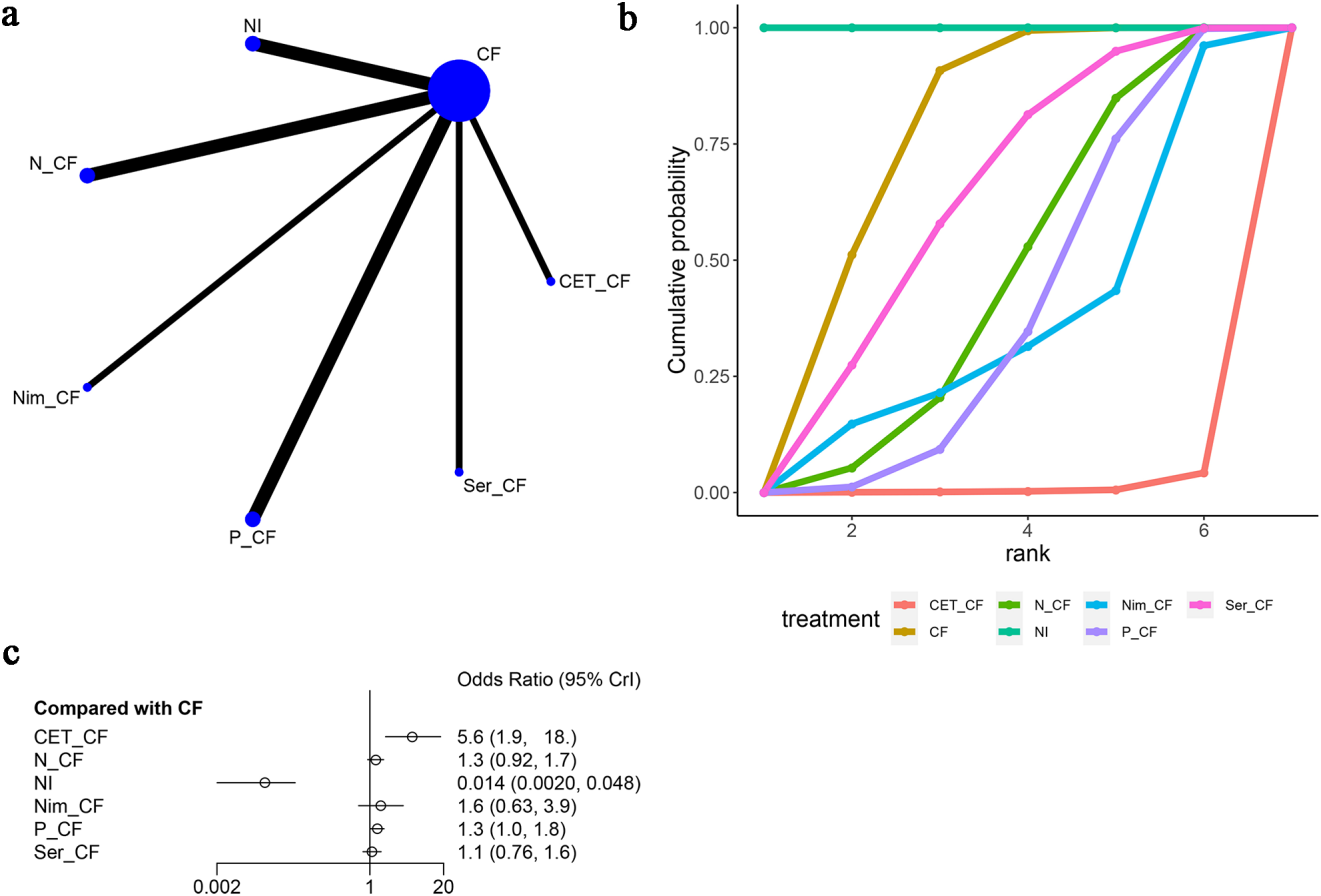
Meta-Analysis of Neutropenia. **(A)** Network Plot(Each circle represents a different intervention, with the size of the circle proportional to the number of people in that intervention, and the line between the circles represents the existence of a direct comparison between the two interventions, with the thickness of the line representing the proportional number of studies), **(B)** Area under the Cumulative Probability Curve (Surface Under the Cumulative Ranking Curve of different intervention, CF, Cisplatin + Fluorouracil; NI, Nivolumab + Ipilimumab,P-CF, Pembrolizumab + Cisplatin + Fluorouracil,CET-CF, Cetuximab + Cisplatin + Fluorouracil,N-CF, Nivolumab + Cisplatin + Fluorouracil,Nim-CF, Nimotuzumab + Cisplatin + Fluorouracil,Ser-CF, Serplulimab + Cisplatin + Fluorouracil), **(C)** Forest Plot.

Seven studies (13, 15–20) reported neutropenia as an adverse event, as illustrated in **Figure 8**. The network diagram (**Figure 8A**) displayed direct comparisons between CF and NI, CF and N-CF, CF and Nim-CF, CF and P-CF, CF and Ser-CF, and CF and CET-CF. Compared to CF, CET-CF [OR=5.6, 95% CI (1.9, 18.0)], N-CF [OR=1.3, 95% CI (0.92, 1.7)], NI [OR=0.014, 95% CI (0.002, 0.048)], Nim-CF [OR=1.6, 95% CI (0.63, 3.9)], P-CF [OR=1.3, 95% CI (1.0, 1.8)], and Ser-CF [OR=1.1, 95% CI (0.76, 1.6)] were analyzed. In comparison to CF, P-CF and CET-CF increased the probability of neutropenia, with CET-CF having the highest likelihood. However, Ser-CF, Nim-CF, NI, and N-CF had a lower probability of causing neutropenia (**Figure 8C**). The area under the cumulative ranking curve indicated that NI had the highest impact (100%), followed by CF (73.6%), Ser-CF (60.2%), and CET-CF (0.9%) (**Figure 8B**; **Table 3**).

**Figure 9:**
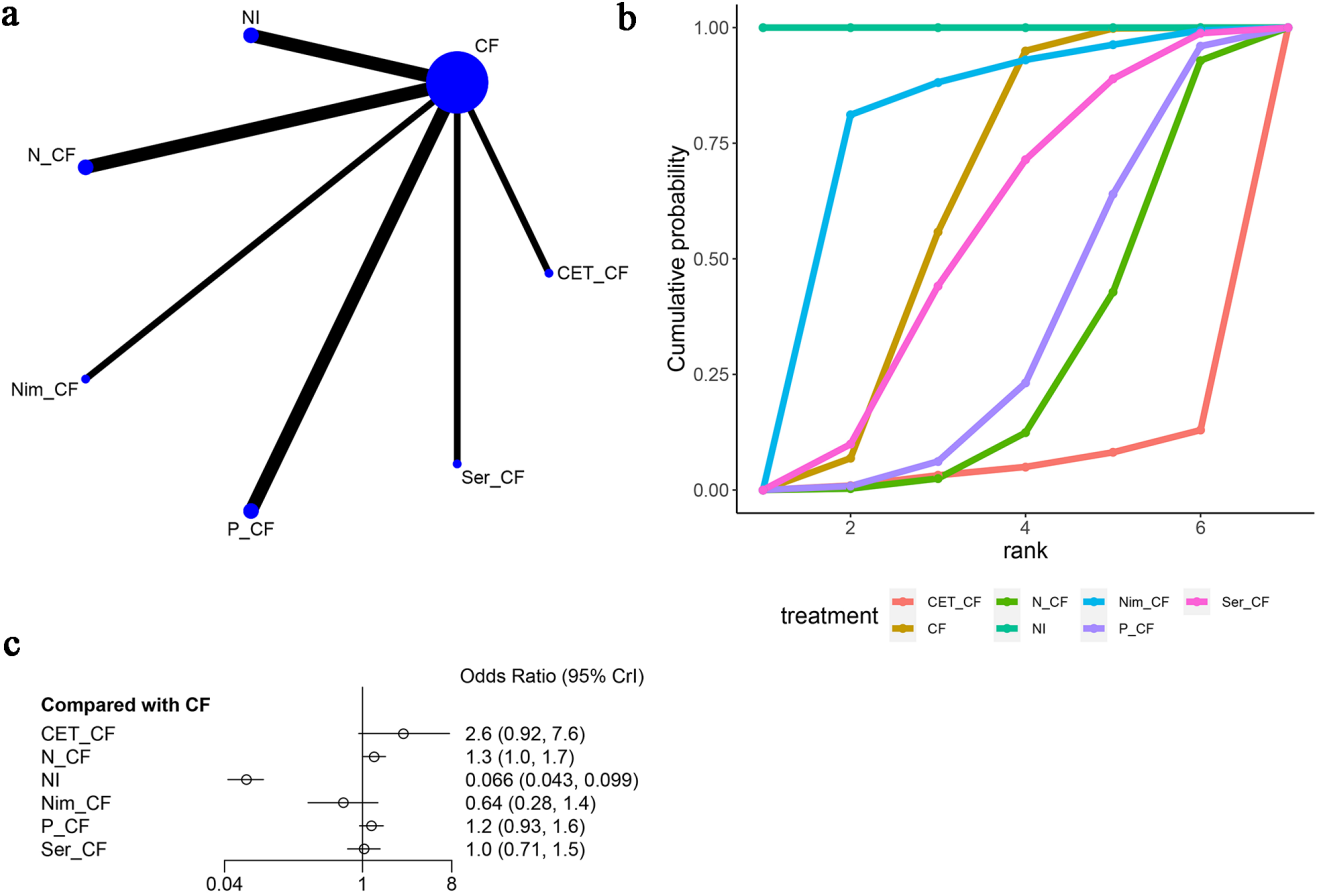
Meta-Analysis of Nausea. **(A)** Network Plot(Each circle represents a different intervention, with the size of the circle proportional to the number of people in that intervention, and the line between the circles represents the existence of a direct comparison between the two interventions, with the thickness of the line representing the proportional number of studies), **(B)** Area under the Cumulative Probability Curve(Surface Under the Cumulative Ranking Curve of different intervention, CF, Cisplatin + Fluorouracil; NI, Nivolumab + Ipilimumab,P-CF, Pembrolizumab + Cisplatin + Fluorouracil,CET-CF, Cetuximab + Cisplatin + Fluorouracil,N-CF, Nivolumab + Cisplatin + Fluorouracil,Nim-CF, Nimotuzumab + Cisplatin + Fluorouracil,Ser-CF, Serplulimab + Cisplatin + Fluorouracil), **(C)** Forest Plot.

Additionally, in these seven articles (13, 15–20), nausea as an adverse event was mentioned, as shown in **Figure 9**. The network diagram (**Figure 9A**) did not form a closed-loop structure between the various interventions. Compared to CF, CET-CF [OR=2.6, 95% CI (0.92, 7.6)], N-CF [OR=1.3, 95% CI (1.0, 1.7)], NI [OR=0.066, 95% CI (0.043, 0.099)], Nim-CF [OR=0.64, 95% CI (0.28, 1.4)], P-CF [OR=1.2, 95% CI (0.93, 1.6)], and Ser-CF [OR=1.0, 95% CI (0.71, 1.5)] were evaluated. Compared to CF, CET-CF and N-CF had an increased probability of causing nausea, with CET-CF having the highest likelihood. Conversely, NI, Nim-CF, P-CF, and Ser-CF had a lower probability of causing nausea, with NI having the lowest probability (**Figure 9C**). The area under the cumulative ranking curve indicated that NI had the highest impact (100%), followed by Nim-CF (76.3%), CF (59.6%), and CET-CF (5%) (**Figure 9B**; **Table 3**).

**Figure 10:**
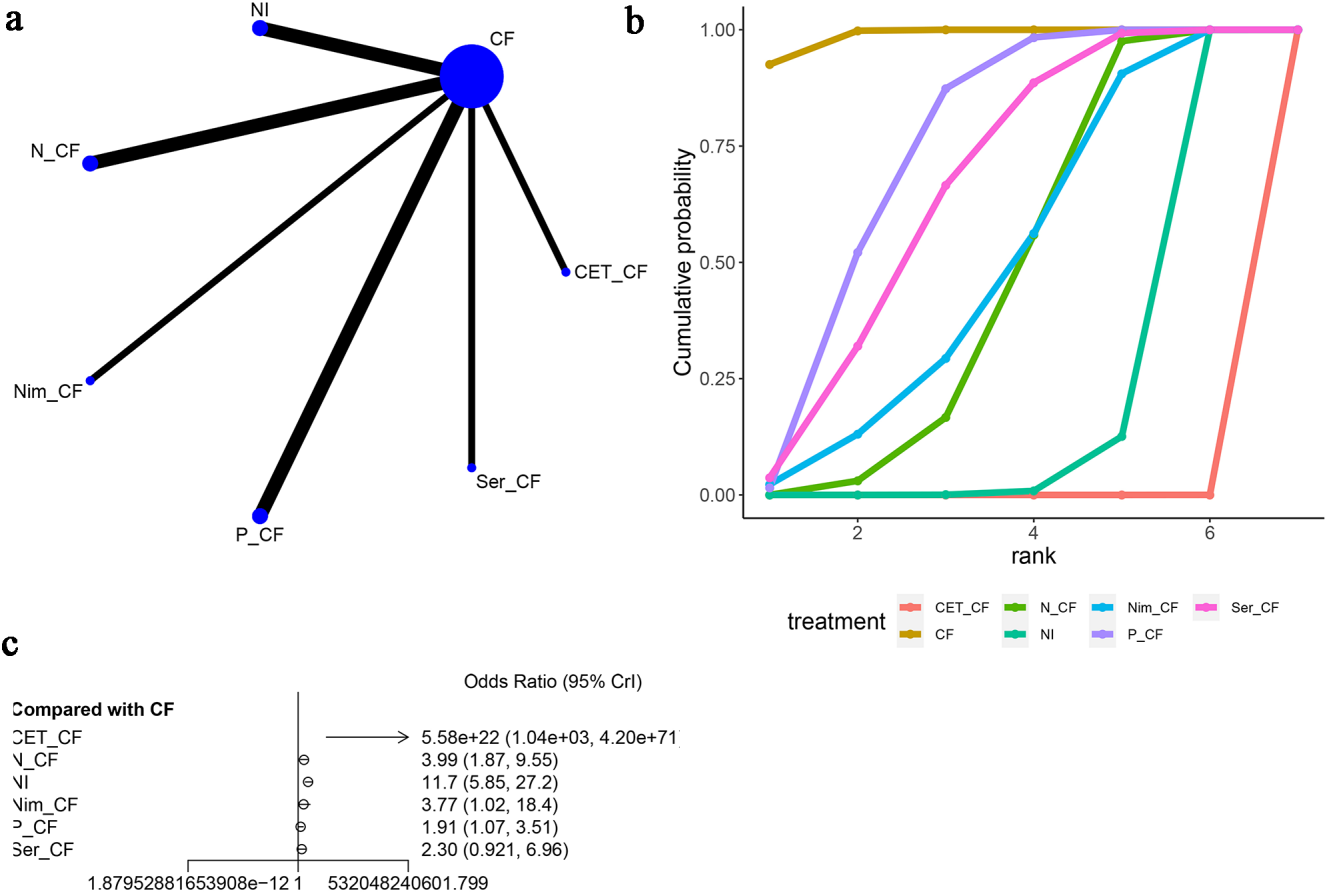
Meta-Analysis of SKIN. **(A)** Network Plot(Each circle represents a different intervention, with the size of the circle proportional to the number of people in that intervention, and the line between the circles represents the existence of a direct comparison between the two interventions, with the thickness of the line representing the proportional number of studies), **(B)** Area under the Cumulative Probability Curve(Surface Under the Cumulative Ranking Curve of different intervention, CF, Cisplatin + Fluorouracil; NI, Nivolumab + Ipilimumab,P-CF, Pembrolizumab + Cisplatin + Fluorouracil,CET-CF, Cetuximab + Cisplatin + Fluorouracil,N-CF, Nivolumab + Cisplatin + Fluorouracil,Nim-CF, Nimotuzumab + Cisplatin + Fluorouracil,Ser-CF, Serplulimab + Cisplatin + Fluorouracil), **(C)** Forest Plot.

Furthermore, skin disorders were mentioned in six articles (13, 15–20), as shown in **Figure 10**. The network diagram (**Figure 10A**) did not form a closed-loop structure between CF and the various interventions. Compared to CF, CET-CF [OR=5.58e+22, 95% CI (1.04e+03, 4.20e+71)], N-CF [OR=3.99, 95% CI (1.87, 9.55)], NI [OR=11.7, 95% CI (5.85, 27.2)], Nim-CF [OR=3.77, 95% CI (1.02, 18.4)], P-CF [OR=1.91, 95% CI (1.07, 3.51)], and Ser-CF [OR=2.30, 95% CI (0.921, 6.96)] were analyzed. It was observed that all interventions had a higher probability of causing skin adverse events compared to CF, with CET-CF having the highest probability and Ser-CF having the lowest probability. The area under the cumulative ranking curve indicated that CF had the highest impact (98.7%), followed by P-CF (73.2%), Ser-CF (65.0%), and CET-CF (0.00%) (**Figure 10B**; **Table 3**).

**Figure 11:**
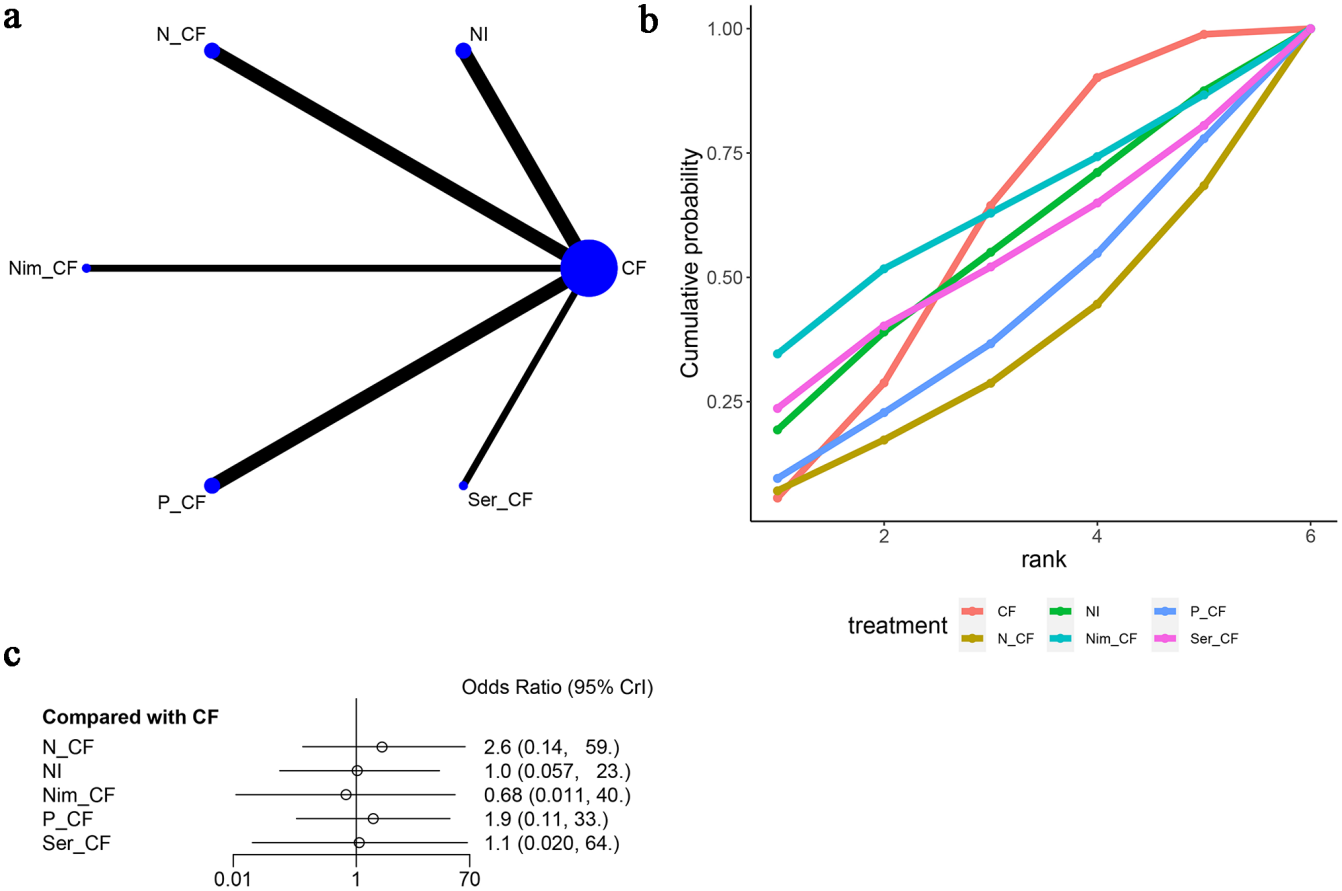
Meta-Analysis of Anorexia. **(A)** Network Plot(Each circle represents a different intervention, with the size of the circle proportional to the number of people in that intervention, and the line between the circles represents the existence of a direct comparison between the two interventions, with the thickness of the line representing the proportional number of studies), **(B)** Area under the Cumulative Probability Curve(Surface Under the Cumulative Ranking Curve of different intervention, CF, Cisplatin + Fluorouracil; NI, Nivolumab + Ipilimumab,P-CF, Pembrolizumab + Cisplatin + Fluorouracil,N-CF, Nivolumab + Cisplatin + Fluorouracil,Nim-CF, Nimotuzumab + Cisplatin + Fluorouracil,Ser-CF, Serplulimab + Cisplatin + Fluorouracil), **(C)** Forest Plot.

Six articles (15–20) reported instances of anorexic adverse reactions, as depicted in **Figure 11**. The network diagram (**Figure 11A**) indicates that the various treatment regimens did not form a closed-loop structure. Direct comparisons exist between CF and NI, CF and N-CF, CF and Nim-CF, CF and P-CF, and CF and Ser-CF. Compared to CF, the odds ratios (OR) and 95% confidence intervals (CI) for anorexic reactions are as follows: N-CF [OR=2.6, 95%CI (0.14, 59.0)], NI [OR=1.0, 95%CI (0.057, 23.0)], Nim-CF [OR=0.68, 95%CI (0.011, 40.0)], P-CF [OR=1.9, 95%CI (0.11, 33.0)], and Ser-CF [OR=1.1, 95%CI (0.020, 64.0)]. From this, it can be inferred that the likelihood of experiencing anorexia is higher with N-CF and lower with Nim-CF (**Figure 11C**). The cumulative ranking probability curves indicate that Nim-CF had the highest area under the curve (62.1%), followed by CF (57.6%), NI (54.4%), and N-CF (33.3%) being the lowest (**Figure 11B**; **Table 3**). Based on these four adverse reactions, it can be concluded that CET-CF has a higher probability of presenting the aforementioned adverse events, while NI has a lower probability.

In summary, based on the four types of adverse reactions mentioned above, CET-CF had a higher probability of causing these adverse events, while NI had a lower probability.

### 3.7 Publication bias assessment

We used funnel plots to assess publication bias for ORR, DCR, and the four discussed adverse reactions in this article. The results indicated a significant likelihood of publication bias for ORR, DCR, and the four adverse reactions (**Suplementary Table S2**, **Suplementary Figures S1**-**S6**).

## 4 Discussion

We have observed that many articles primarily focus on comparing two treatment regimens or evaluating the efficacy and safety of neoadjuvant therapy regimens, with most of them comparing two different interventions (21, 22). This study, for the first time, evaluates the efficacy and safety of various first-line treatment approaches for esophageal cancer, marking the novelty of our research.

Our study did not find a significant improvement in overall survival (OS) and progression-free survival (PFS) when comparing different intervention measures with CF (Cisplatin + fluorouracil) as the reference. According to the SUCRA(Surface Under the Cumulative Ranking) ranking, it appeared that Ser-CF (Serplulimab + Cisplatin + fluorouracil) might have the most favorable effects on OS and PFS, followed by P-CF (Pembrolizumab + Cisplatin + fluorouracil). In terms of objective response rate (ORR), N-CF (Nivolumab + Cisplatin + fluorouracil) significantly improved ORR, while CET-CF (Cetuximab + cisplatin + fluorouracil) notably extended disease control rate (DCR). However, CET-CF was associated with a higher probability of adverse effects such as nausea, vomiting, neutropenia, and rash.

Some studies have demonstrated that cetuximab in combination with standard chemotherapy can significantly improve the PFS and OS of esophageal cancer patients (23). A possible explanation, based on literature review, could be that CET might have negative interactions with platinum-based drugs, possibly limiting their ability to induce oxidative damage (24). In line with most articles, cetuximab can enhance ORR and DCR in esophageal cancer patients (25), but it has been noted that its use is associated with a higher likelihood of rash (24). Nimotuzumab, when combined with CF treatment, has shown promising antitumor activity and good tolerability (4). Cetuximab and nimotuzumab are both EGFR antibodies that inhibit tumor cell growth, angiogenesis, and apoptosis. However, nimotuzumab’s unique property of requiring bivalent binding for stable attachment to cell surfaces potentially confers greater clinical benefit and is not associated with severe skin toxicity (4). Possible reasons for the lack of significant improvement in OS and PFS for the two targeted drugs mentioned in this article may include the influence of various molecular factors, such as EGFR gene copy number, KRAS mutations, AKT, ERK, and other biomarkers (26). Previous studies suggested that cetuximab may be ineffective in patients with low EGFR expression, and resistance to EGFR-targeted therapy can arise due to EGFR signaling-related gene mutations (27).

Comparing CF and CD (cisplatin + docetaxel) regimens, it has been reported that CD did not improve the OS and PFS of esophageal cancer patients in the final concurrent chemoradiotherapy (CCRT) (21). This suggests that CF remains the current standard chemotherapy regimen. One of the most significant advancements in the treatment of cancer was immunotherapy, particularly immune checkpoint inhibitors(ICIs).As mentioned above, Ser-CF (Serplulimab + Cisplatin + fluorouracil) might have the most favorable effects on OS and PFS. Some studies also have demonstrated that Serplulimab had the highest probability for better OS and PFS in other solid tumors (28). Serplulimab is a novel humanized anti-PD-1 monoclonal antibody and it has high receptor occupancy and strong target action level. Compared with other ICIs in the paper, Serplulimab had no antibody-dependent cell-mediated cytotoxicity(ADCC) and complement dependent cytotoxicity(CDC) effects and could avoid the elimination of T cells, so that it can fully kill tumor cells and play a better curative effect (29, 30). Evidence indicates that intrinsic factors of tumor cells, such as PD-L1 expression, tumor mutational burden, and microsatellite instability-high status, are associated with the efficacy of immune checkpoint inhibitors (31). In the KEYNOTE-180 trial, it was demonstrated that patients with high PD-L1 expression (CPS ≥ 10) had higher OS rates than those with low PD-L1 expression (31). Several articles have analyzed that for esophageal squamous cell carcinoma (ESCC) patients with low or negative PD-L1 expression, anti-PD-L1 treatment may not provide a survival advantage (32–34), and the incidence of treatment-related adverse events and ≥Grade 3 adverse events was lower in the immune checkpoint inhibitor (ICI) group (35).

In this article, differences among the top-ranked interventions combining immunotherapy and chemotherapy do not appear to be significant. This may be attributed to the lack of subgroup analysis based on PD-L1 expression levels in the network meta-analysis. Upon reviewing the included original articles, it was reasonable that a considerable portion of the total population had low PD-L1 expression. This aligns with the conclusion from related literature that adding ICI to esophageal cancer patients with low PD-L1 expression does not confer benefits. Therefore, it can be inferred that the lack of significant differences among these intervention measures is likely due to the inclusion of a substantial number of patients with low PD-L1 expression.

It is essential to acknowledge the limitations of this article. Firstly, we did not conduct a subgroup analysis of PD-L1 expression levels among the included studies but evaluated all patients as a whole, leading to limited variation in the results. Secondly, we did not perform subgroup analysis based on histological types of esophageal cancer.

## 5 Conclusion

Based on the current research, it can be concluded that all of the above measures contribute to the improvement of Overall Survival (OS) and Progression-Free Survival (PFS). Among these, the Ser-CF regimen appears to be the most effective in enhancing OS and PFS outcomes, while N-CF significantly extends Objective Response Rate (ORR), and CET-CF notably prolongs Disease Control Rate (DCR). It is important to note that CET-CF is associated with a higher probability of adverse events such as nausea, vomiting, neutropenia, and skin rash.
