# Supplementary material for: Efficacy and safety evaluation of first-line systemic treatments for unresectable esophageal squamous cell carcinoma: a network meta-analysis
Source: Front Oncol. 2024 Sep 9;14:1397960. doi: 10.3389/fonc.2024.1397960 (PMC11416913; doi:10.3389/fonc.2024.1397960)
Supplement: Supplementary file 1 [file DataSheet1.pdf]

Efficacy and Safety Evaluation of First-Line Systemic Treatments for Unresectable Esophageal Squamous Cell Carcinoma: A Network Meta-Analysis

keywords:

- First-line treatment
- Advanced esophageal squamous carcinoma
- Network meta-analysis
- Efficacy
- Safety

The first-line treatment regimens

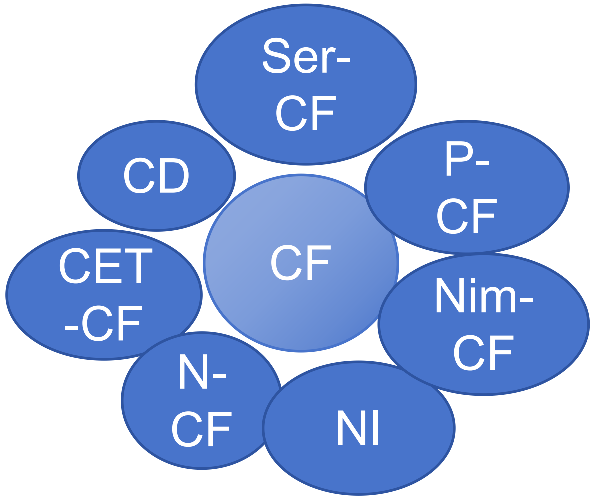

Outcomes

- Overall Survival(OS)
- Progression-Free Survival(PFS)
- Objective Response Rate(ORR)
- Disease Control Rate(DCR)
- Adverse Effects(AEs)

CF: Cisplatin + 5-fluorouracil; P-CF: Pembrolizumab+ Cisplatin + 5-fluorouracil; N-CF: Nivolumab+ Cisplatin+ fluorouracil; Nim-CF: Cisplatin+ fluorouracil+ nimotuzumab; Ser-CF: Serplulimab+ Cisplatin + 5-fluorouracil; CET-CF: Cetuximab+ cisplatin+ 5-fluorouracil; NI: Nivolumab+ Ipilimumab; CD: Cisplatin+ docetaxel;
