# Supplementary material for: Efficacy and safety evaluation of first-line systemic treatments for unresectable esophageal squamous cell carcinoma: a network meta-analysis
Source: Front Oncol. 2024 Sep 9;14:1397960. doi: 10.3389/fonc.2024.1397960 (PMC11416913; doi:10.3389/fonc.2024.1397960)
Supplement: Supplementary file 2 [file DataSheet2.docx]

**TableS1. Search Strategy**

1. Pubmed

| ((("Esophageal Neoplasms"[Mesh]) OR ((((((((((((((((((Esophageal Neoplasms[Title/Abstract]) OR (Esophageal Neoplasm[Title/Abstract])) OR (Neoplasm, Esophageal[Title/Abstract])) OR (Esophagus Neoplasm[Title/Abstract])) OR (Esophagus Neoplasms[Title/Abstract])) OR (Neoplasm, Esophagus[Title/Abstract])) OR (Neoplasms, Esophagus[Title/Abstract])) OR (Neoplasms, Esophageal[Title/Abstract])) OR (Cancer of Esophagus[Title/Abstract])) OR (Cancer of the Esophagus[Title/Abstract])) OR (Esophagus Cancer[Title/Abstract])) OR (Cancer, Esophagus[Title/Abstract])) OR (Cancers, Esophagus[Title/Abstract])) OR (Esophagus Cancers[Title/Abstract])) OR (Esophageal Cancer[Title/Abstract])) OR (Cancer, Esophageal[Title/Abstract])) OR (Cancers, Esophageal[Title/Abstract])) OR (Esophageal Cancers[Title/Abstract]))) AND (((((newly diagnosed[Title/Abstract]) OR (untreated[Title/Abstract])) OR (first-line[Title/Abstract])) OR (front-line[Title/Abstract])) OR (initial[Title/Abstract]))) AND (randomized controlled trial[Publication Type] OR randomized[Title/Abstract] OR placebo[Title/Abstract]) |
| --- |

2. Embase

| No. | Query | Results |
| --- | --- | --- |
| #31 | #20 AND #26 AND #30 | 183 |
| #30 | #27 OR #28 OR #29 | 867271 |
| #29 | 'double-blind':ab,ti | 226370 |
| #28 | 'placebo':ab,ti | 368441 |
| #27 | 'random':ab,ti | 439439 |
| #26 | #21 OR #22 OR #23 OR #24 OR #25 | 1851542 |
| #25 | 'initial':ab,ti | 1312109 |
| #24 | 'front-line':ab,ti | 14235 |
| #23 | 'first-line':ab,ti | 193446 |
| #22 | 'untreated':ab,ti | 277559 |
| #21 | 'newly diagnosed':ab,ti | 119873 |
| #20 | #1 OR #2 OR #3 OR #4 OR #5 OR #6 OR #7 OR #8 OR #9 OR #10 OR #11 OR #12 OR #13 OR #14 OR #15 OR #16 OR #17 OR #18 OR #19 | 114179 |
| #19 | 'esophageal cancers':ab,ti | 2605 |
| #18 | 'cancers, esophageal':ab,ti | 94 |
| #17 | 'cancer, esophageal':ab,ti | 716 |
| #16 | 'esophageal cancer':ab,ti | 35458 |
| #15 | 'esophagus cancers':ab,ti | 85 |
| #14 | 'cancers, esophagus':ab,ti | 34 |
| #13 | 'cancer, esophagus':ab,ti | 114 |
| #12 | 'esophagus cancer':ab,ti | 830 |
| #11 | 'cancer of the esophagus':ab,ti | 1787 |
| #10 | 'cancer of esophagus':ab,ti | 129 |
| #9 | 'neoplasms, esophageal':ab,ti | 17 |
| #8 | 'neoplasms, esophagus':ab,ti | 3 |
| #7 | 'neoplasm, esophagus':ab,ti | 4 |
| #6 | 'esophagus neoplasms':ab,ti | 10 |
| #5 | 'esophagus neoplasm':ab,ti | 2 |
| #4 | 'neoplasm, esophageal':ab,ti | 8 |
| #3 | 'esophageal neoplasm':ab,ti | 216 |
| #2 | 'esophageal neoplasms':ab,ti | 512 |
| #1 | 'esophagus tumor'/exp | 111219 |

3. Web of science

| # |  | result |
| --- | --- | --- |
| 1 | TS=(Esophageal Neoplasms) OR TS=(Esophageal Neoplasm) OR TS=(Neoplasm, Esophageal) OR TS=(Esophagus Neoplasm) OR TS=(Esophagus Neoplasms) OR TS=(Neoplasm, Esophagus) OR TS=(Neoplasms, Esophagus) OR TS=(Neoplasms, Esophageal) OR TS=(Cancer of Esophagus) OR TS=(Cancer of the Esophagus) OR TS=(Esophagus Cancer) OR TS=(Cancer, Esophagus) OR TS=(Cancers, Esophagus) OR TS=(Esophagus Cancers) OR TS=(Esophageal Cancer) OR TS=(Cancer, Esophageal) OR TS=(Cancers, Esophageal) OR TS=(Esophageal Cancers) | 63850 |
| 2 | TS=(newly diagnosed) OR TS=(untreated) OR TS=(first-line) OR TS=(front-line) OR TS=(initial) | 2219608 |
| 3 | TS=(random) OR TS=(placebo) OR TS=(double-blind) OR TS=(randomized controlled trial) OR TS=(randomized) | 2260761 |
| 4 | #3 AND #2 AND #1 | 469 |

**TableS2.League table**

1. Neutropenia League Table

| OR(95%Crl) | | | | | | |
| --- | --- | --- | --- | --- | --- | --- |
| CET_CF |  |  |  |  |  |  |
| 5.56 (1.9, 17.69) | CF |  |  |  |  |  |
| 4.41 (1.43, 14.66) | 0.79 (0.57, 1.09) | N_CF |  |  |  |  |
| 413.23 (74.96, 3724.35) | 71.77 (20.84, 496.9) | 90.59 (25.15, 639.62) | NI |  |  |  |
| 3.57 (0.86, 15.34) | 0.64 (0.26, 1.58) | 0.81 (0.31, 2.09) | 0.01 (0, 0.04) | Nim_CF |  |  |
| 4.14 (1.36, 13.66) | 0.75 (0.56, 0.99) | 0.94 (0.61, 1.44) | 0.01 (0, 0.04) | 1.16 (0.45, 3.02) | P_CF |  |
| 5.09 (1.63, 17.17) | 0.92 (0.64, 1.32) | 1.16 (0.71, 1.89) | 0.01 (0, 0.05) | 1.43 (0.54, 3.81) | 1.23 (0.77, 1.95) | Ser_CF |

2. Nausea League Table

| OR(95%Crl) | | | | | | |
| --- | --- | --- | --- | --- | --- | --- |
| CET_CF |  |  |  |  |  |  |
| 2.58 (0.92, 7.58) | CF |  |  |  |  |  |
| 1.98 (0.68, 5.99) | 0.77 (0.59, 1) | N_CF |  |  |  |  |
| 39.09 (12.82, 123.17) | 15.08 (10.11, 23.12) | 19.72 (12.16, 32.55) | NI |  |  |  |
| 4.05 (1.09, 15.65) | 1.57 (0.7, 3.56) | 2.04 (0.87, 4.85) | 0.1 (0.04, 0.26) | Nim_CF |  |  |
| 2.1 (0.72, 6.36) | 0.81 (0.62, 1.07) | 1.06 (0.72, 1.56) | 0.05 (0.03, 0.09) | 0.52 (0.22, 1.22) | P_CF |  |
| 2.5 (0.83, 7.8) | 0.97 (0.67, 1.42) | 1.26 (0.79, 2.01) | 0.06 (0.04, 0.11) | 0.62 (0.25, 1.51) | 1.19 (0.75, 1.89) | Ser_CF |

3. Skin Disorders League Table

| OR(95%Crl) | | | | | |
| --- | --- | --- | --- | --- | --- |
| CF |  |  |  |  |  |
| 0.25 (0.1, 0.54) | N_CF |  |  |  |  |
| 0.09 (0.04, 0.17) | 0.34 (0.11, 1.04) | NI |  |  |  |
| 0.26 (0.05, 0.98) | 1.04 (0.17, 5.13) | 3.11 (0.52, 14.81) | Nim_CF |  |  |
| 0.52 (0.28, 0.94) | 2.09 (0.78, 5.94) | 6.17 (2.4, 17.14) | 1.99 (0.47, 11.28) | P_CF |  |
| 0.43 (0.14, 1.08) | 1.71 (0.44, 6.18) | 5.08 (1.34, 17.58) | 1.63 (0.3, 10.45) | 0.82 (0.23, 2.5) | Ser_CF |

4. Anorexia League Table

| OR(95%Crl) | | | | | |
| --- | --- | --- | --- | --- | --- |
| CF |  |  |  |  |  |
| 0.38 (0.02, 7.4) | N_CF |  |  |  |  |
| 0.96 (0.04, 17.52) | 2.51 (0.04, 171.57) | NI |  |  |  |
| 1.48 (0.02, 90.48) | 3.9 (0.03, 683.04) | 1.54 (0.01, 271.63) | Nim_CF |  |  |
| 0.53 (0.03, 9.35) | 1.39 (0.02, 97.04) | 0.56 (0.01, 37.36) | 0.36 (0, 52.13) | P_CF |  |
| 0.9 (0.02, 48.92) | 2.33 (0.02, 377.29) | 0.93 (0.01, 154.66) | 0.6 (0, 193) | 1.67 (0.01, 236.07) | Ser_CF |


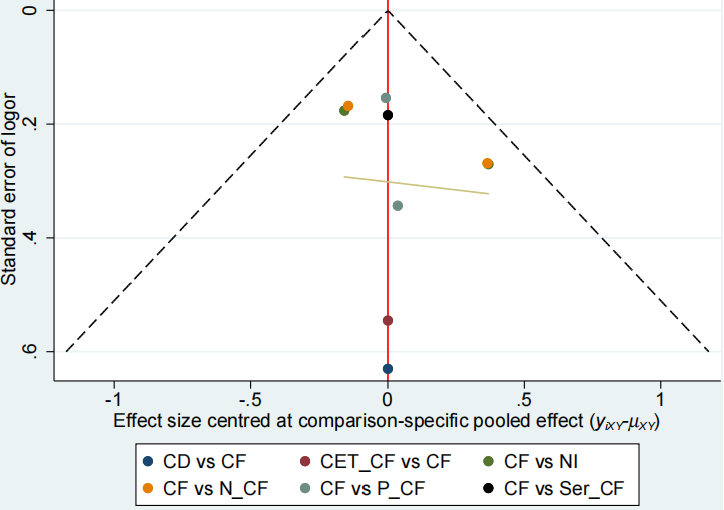


FigureS1.ORR Funnel Plot


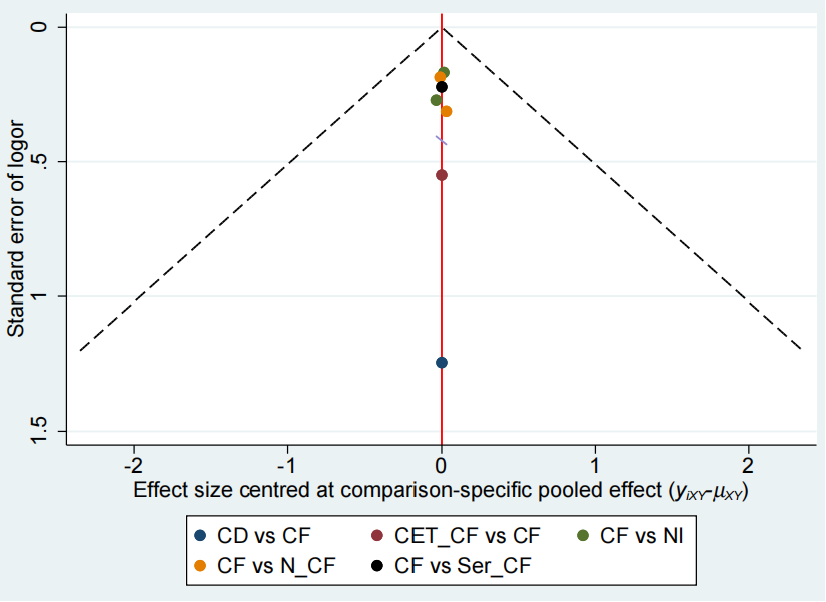


FigureS2.DCR Funnel Plot


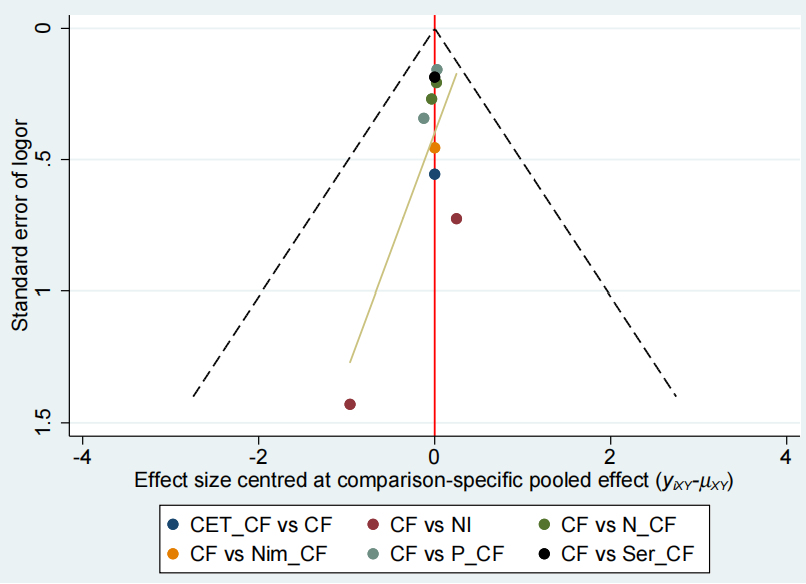


FigureS3.Neutropenia Funnel Plot


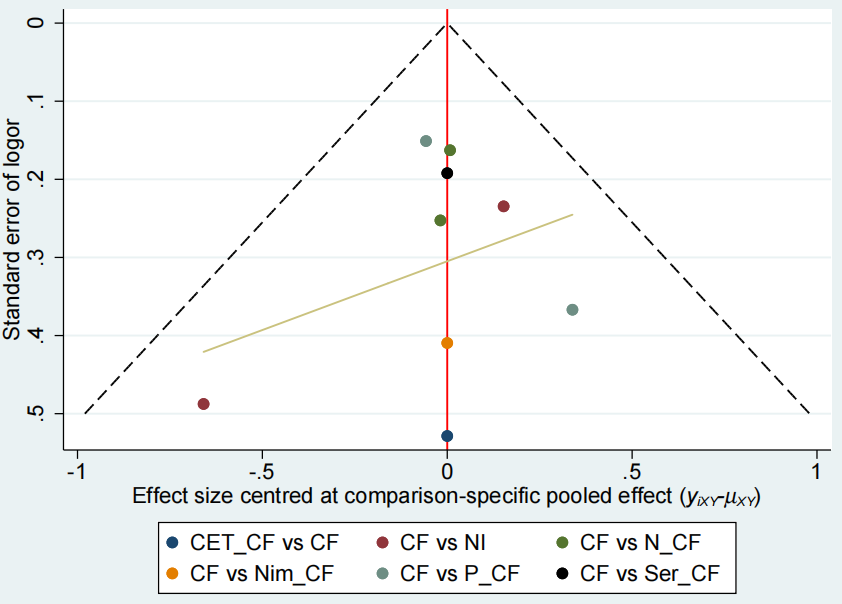


FigureS4.Nausea Funnel Plot


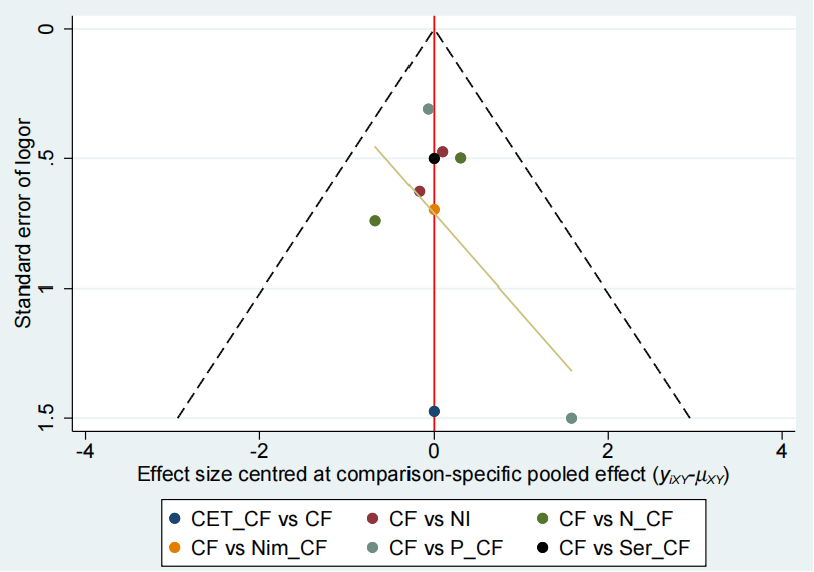


FigureS5.Skin disorders Funnel Plot


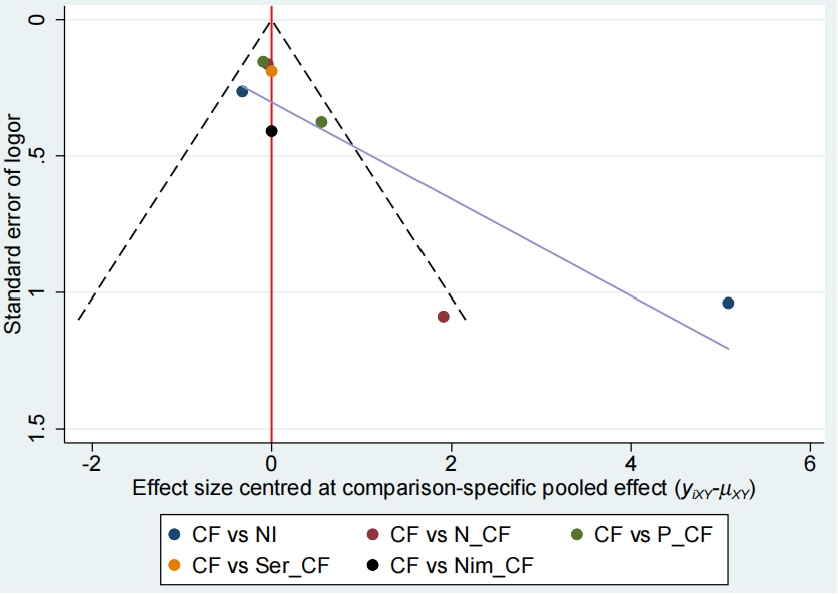


FigureS6.Anorexia Funnel Plot
