# Supplementary material for: Efficacy and safety evaluation of first-line systemic treatments for unresectable esophageal squamous cell carcinoma: a network meta-analysis
Source: Front Oncol. 2024 Sep 9;14:1397960. doi: 10.3389/fonc.2024.1397960 (PMC11416913; doi:10.3389/fonc.2024.1397960)
Supplement: Supplementary file 3 [file DataSheet3.docx]

**Appendix 1: R code**

install.packages('gemtc')

install.packages('ggplot2')

**Load packet**

setwd('D:\\Working directory ')

library(gemtc)

library(coda)

**Read data and transform data**

data <- read.csv('D:\\Working directory\\Workbook 1.csv')

network <- mtc.network(data)

Network Mapping

plot(network)

**Consistency modeling**

model<-mtc.model(network,type="consistency",n.chain=4,likelihood="normal",link="identity",linearModel="random")

**Model iteration**

results <- mtc.run(model, n.adapt = 20000, n.iter = 50000, thin = 1)

summary(results)

**Inconsistency test**

modelume<-mtc.model(network,type="ume",n.chain=4,likelihood="normal",link="identity",linearModel="random")

**Model iteration**

resultsme <- mtc.run(model, n.adapt = 20000, n.iter = 50000, thin = 1)

summary(resultsme)

**Forest map**

forest(results)

forest(relative.effect(results, "Placebo"),digits=3,xlim = c(-3,1))

ranks <- rank.probability(results,preferredDirection= -1)

plot(ranks)

plot(ranks,beside = TRUE)

sucra(ranks)

write.csv(ranks,"TFranks.csv")

library(ggplot2)

TFrank<- read.csv('D:\\Working directory\\TFranks.csv')

tb<- relative.effect.table(results)

tb1<-round((tb),2)

write.csv(tb1,"HbA1c_table.csv")

resultanohe <- mtc.anohe(network)

c<-summary(resultanohe)

print(c)
